# Mitral degenerative disease mimicking a valvular tumor: a case report

**DOI:** 10.1186/s13019-015-0345-3

**Published:** 2015-11-02

**Authors:** Eiki Tayama, Tomohiro Ueda, Ryusuke Mori, Ken-ichi Imasaka, Yukihiro Tomita

**Affiliations:** Department of Cardiovascular Surgery, Clinical Research Institute, Kyushu Medical Center, National Hospital Organization, Fukuoka, Japan

**Keywords:** Mitral valve, Valvular tumor, Valvular mass, Degenerative disease, Echocardiography

## Abstract

**Background:**

In rare cases, echo findings of degenerative valve disease is similar to valvular mass.

**Case Presentation:**

A 56-year-old woman was evaluated for palpitation. Echocardiography revealed an 8- mm mass on the anterior mitral leaflet with minimal mitral insufficiency. Resection of the valve tumor was attempted to prevent a possible embolism. However, the lesion was not a tumor, but an aneurysm-like bulge on the anterior leaflet without chorda elongation. Triangular resection and ring annuloplasty were performed. The patient’s postoperative course was uneventful. Pathological examination revealeddegenerative disease.

**Conclusions:**

This case illustrates that a valvular mass that looks like a tumor by echocardiography may actually be degenerative regardless of the presence of mitral insufficiency.

## Background

When a mass is observed on the mitral valve by echocardiography, infectious vegetation and various types of valve tumors are generally suspected [1–6]. However, degenerative disease also occasionally looks like a valvular mass [6]. Diagnosis of degenerative disease is not usually difficult because it is characterized by flail leaflet motion with concomitant regurgitation. If the diseased lesion is limited in size and regurgitation is not significant, however, diagnosis becomes more difficult. We herein report a case of mitral degenerative disease without regurgitation that looked like a valvular tumor.

## Case presentation

**Fig 1:**
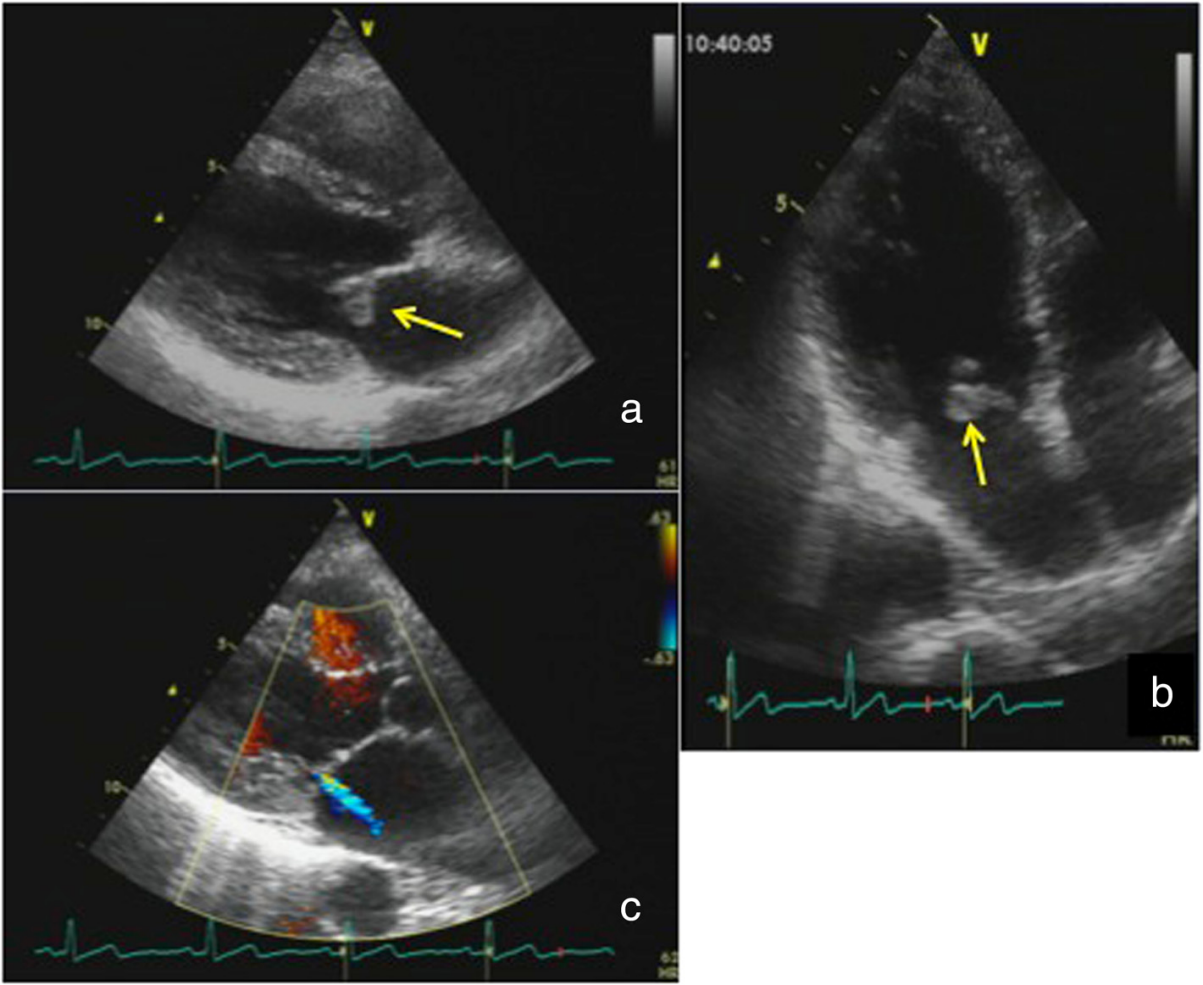
Echocardiographic findings. An 8- × 8-mm tumor-like lesion (↑) was present on the posterior side of the anterior mitral leaflet with trivial regurgitation. **a**, **c** Long-axis view. **b** Four-chamber view

**Fig 2:**
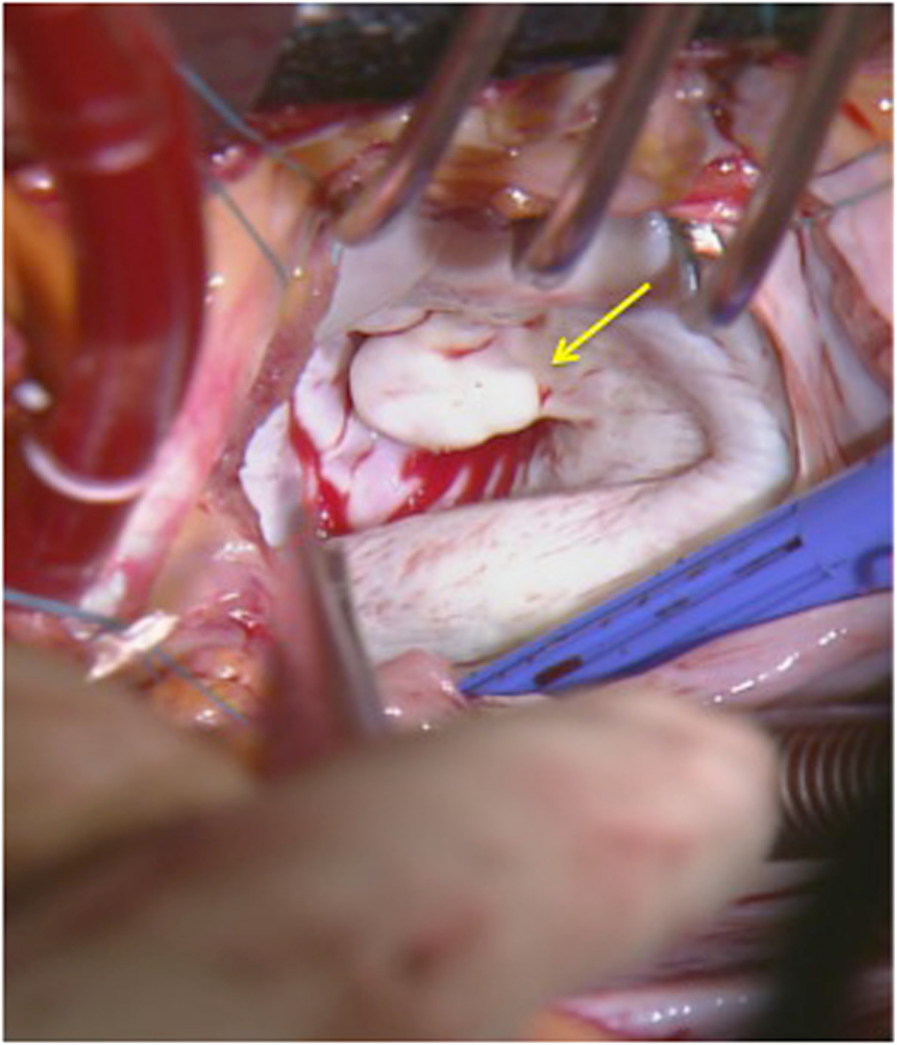
Intraoperative findings (operator’s view). An aneurysm-like bulge was found on the posterior side of the anterior mitral leaflet

A 56-year-old woman with no infectious history was evaluated for palpitation. She had regular pulse rhythm and no heart murmur. Electrocardiography, Holter, electrocardiography, and chest X-ray findings were normal. Transthoracic echocardiography revealed an 8- × 8-mm mass on the posterior side of the anterior mitral leaflet with minimal mitral insufficiency (Fig. 1). The left ventricular motion and dimensions were normal. Transesophageal echocardiography demonstrated similar findings. Computed tomography showed no systemic embolism. Because a valve tumor was suspected, surgical resection was attempted to prevent a possible systemic embolism. During surgery, we found that the lesion was not a tumor, but an aneurysm-like bulge on the anterior leaflet without chorda elongation (Fig. 2). Triangular resection and ring annuloplasty were performed, and the patient’s postoperative course was uneventful. Pathological examination showed myxomatous change, fibrosis, and hyalinization, confirming a diagnosis of degenerative disease. Now, her condition has been good without any mitral valve abnormality including regurgitation at postoperative 10 months.

## Discussion

When a mass is found on the mitral valve by echocardiography, an infectious vegetation is usually suspected first. Various types of valve tumors may be suspected less frequently. Primary cardiac tumors are uncommon, accounting for 0.021 % among 1 million autopsies [1]. Primary “valve” tumors are rarer, constituting less than 10 % of all primary cardiac tumors [2, 3]. Despite their low incidence, valve tumors are clinically very important because they occasionally become symptomatic, causing serious neurological symptoms or sudden death; this is more common with mitral than aortic valve tumors [4]. The most common histological type of valve tumor is papillary fibroelastoma, followed by myxoma, fibroma, sarcoma, and others [4]. If a valve mass is a highly mobile and large, surgical resection is recommended regardless of the histological findings or presence of symptoms [4–7].

In contrast, degenerative mitral valve disease, such that caused by fibroelastic deficiency, is a common disorder affecting approximately 2 % of the population [8]. Degenerative valve disease occasionally looks like a valve mass [6]. In patients with degenerative valve disease, fibrin deficiency often leads to rupture of one or more thinned and elongated chordae, those often associated with the middle scallop of the posterior leaflet. As a result, most patients with degenerative mitral valve disease develop leaflet prolapse, resulting in varying degrees of regurgitation due to leaflet malcoaptation during ventricular contraction. However, if chordal damage is minimal despite the presence of myxomatous change in the leaflet, valve regurgitation can be minimal. In such cases, distinction between a tumor and degenerative disease is difficult [6].

## Conclusion

In conclusion, an aneurysm-like bulge on the leaflet without major regurgitation was misdiagnosed as a valve tumor in this case; the patient actually had degenerative disease. Our assumption that degenerative disease should coexist with regurgitation led to misdiagnosis. Our experience serves to remind clinicians that when a valvular mass looks like a tumor by echocardiography, it may instead be a degenerative change, even without mitral insufficiency.

## Consent

Written informed consent was obtained from the patient for publication of this Case Report and any accompanying images. A copy of the written consent is available for review by the Editor-in-Chief of this journal. The ethics committee of our institution approved to publish this case report.
